# Parameter Optimization for Laser Peen Forming on 6005A-T6 Aluminum Alloy Plates to Enhance the Constrained Deformation of Integral Stiffened Plates

**DOI:** 10.3390/ma17205090

**Published:** 2024-10-18

**Authors:** Gaoqiang Jiang, Jianzhong Zhou, Jian Wu, Shu Huang, Xiankai Meng, Yongxiang Hu

**Affiliations:** 1 School of Mechanical Engineering, Jiangsu University, Zhenjiang 212013, China; 2112203011@stmail.ujs.edu.cn (G.J.); huangshu11@sina.com (S.H.); mengdetiankong10@126.com (X.M.); 2 School of Mechanical and Information Engineering, Wuxi Vocational Institute of Arts & Technology, Wuxi 214206, China; xiaowujian4540@163.com; 3 School of Mechanical Engineering, Shanghai Jiao Tong University, Shanghai 200240, China; huyx@sjtu.edu.cn

**Keywords:** laser peen forming, parameter optimization, 6005A-T6 aluminum alloy plates, integral stiffened plate, constrained deformation

## Abstract

Multiscale parameter optimization for laser peen forming (LPF) on 6005A-T6 aluminum alloy plates was conducted through a combination of simulation and experimentation. By obtaining the optimal parameter, this study aims to explore the constrained deformation and forming laws of the integral stiffened plates. Detailed descriptions were provided regarding the dynamic response process and transient behavior of aluminum alloy plates under ultrahigh strain rates, along with an in-depth analysis of the stress evolution. The results reveal that laser beam diameter and laser beam energy can achieve large range forming, while the number of tracks facilitates the precise deformation adjustment. During the 12-track LPF process, there is an overall upward trend in deformation values accompanied by a dynamic increase in the bend curvature. After static relaxation, the deformation value recovers to 55.2% of the final bending curvature. The chord direction scanning of stiffened plates exhibits a larger bending curvature, indicating its greater forming capacity for large-sized single unfolding direction formation; whereas, the unfolding direction scanning of stiffened plates excels in achieving efficient integrated two-way forming.

## 1. Introduction

Currently, the lightweight and high-strength integral components required to maintain the structural integrity of high-speed transport equipment, such as aircraft wings and high-speed rail skins, primarily rely on conventional mechanical bending and shot peening methods [1,2,3]. However, the limitations of these formed components include poor accuracy, thin compressive stress layer, and severe stress concentration issues, which constrain the application of integral stiffened plates (SP) in terms of their formability [4,5,6]. In comparison with other traditional surface strengthening techniques, laser peening (LP) technology offers several advantages, including higher shockwave pressure (GPa), greater power density (GW/cm), increased high strain rate (10^7^/s), and shorter pulse duration (ns) [7,8,9]. When the peak pressure of the laser-induced shockwave surpasses the dynamic yield strength of the materials, plastic deformation transpires in the strengthening zone, producing compressive stress perpendicular to the surface [10,11]. This disrupts the tension–compression stress balance in overall stress fields, leading to stress redistribution for rebalancing purposes [12,13,14]. In order to fully utilize the overall forming effect caused by plastic deformation and stress redistribution to obtain the desired shaped parts, laser peen forming (LPF) technology emerges as a timely solution, with its precise processing method characterized by low cold work hardening, high compressive stress intensity, and processing flexibility, LPF holds promising prospects for broad applications [15,16,17]. The aircraft wings and high-speed rail skins are prone to fatigue damage due to long-term exposure to complex loads. The aircraft skin and wings are treated with LPF to achieve the uniform distribution of the residual stresses on the surface by optimizing laser parameters, significantly improving fatigue resistance.

The key technical challenge of LPF lies in establishing the response law between the target shape, including both plate and SP, and process parameters, such as laser settings, beam size, and control system path. This enables precise controlled forming to be achieved [18]. Hence, numerous experts and scholars have conducted profound and meaningful exploration on the plastic forming characteristics of LPF. Zhang et al. conducted experimental studies to explore the correlation of overall deformation and residual stress field change in aluminum alloy plates of varying geometric thickness [19]. Hu et al. discussed the bending deformation behavior from convex to concave by adjusting laser intensity and plate thickness, respectively [20]. Li et al. examined the influence of pulse duration and pressure distribution in LP on the residual stress field and the formation of holes [21]. However, previous research has primarily focused on the flexible forming characteristics of components using a single parameter, demonstrating the feasibility and application potential of replacing traditional mechanical shot peening with LPF. However, practical problems often involve multiple parameters. Single parameter optimization cannot capture the complex interactions between multiple parameters, nor can it guarantee the globally optimal combination of parameters. This research conducted a thorough investigation into the bend forming of 6005A–T6 aluminum alloy plates by LPF under the cumulative impact of multidimensional parameters to explore the constrained deformation of integral skin structure. Multiparameter optimization research comprehensively considers the interaction of parameters and finds the global optimal solution, which is more in line with practical applications. Reasonable parameter selection can significantly improve the fatigue life of integral skin structures in fields such as aerospace and automotive. Under different parameters, the dynamic response behavior of materials varies significantly, which directly affects the forming effect. Through exploring the parameter optimization and dynamic response of LPF for the integral skin structure, uniform processing can be applied to different positions of this structure. This has a guiding significance for obtaining uniform stress distribution and avoiding stress concentration, thereby significantly improving mechanical properties.

Yang et al. used the average induced stress obtained from representative element models to calculate the saturated bending curvature and improve its computational efficiency in addressing the impact of LP-induced compressive stress on wing forming and fatigue resistance [22]. Glaser et al. optimized the engineering design and used the incremental drilling method to measure residual stress. They found a correlation between Almen deformation amplitude and residual stress, and residual compressive stress can improve the fatigue resistance of aviation components [23]. Cai et al. proposed a feature strain method to reconstruct residual stress fields using the eigenstrain method based on bicubic spline interpolation surface and finite element method, which describes the residual stress distribution characteristics of aviation components [24]. Currently, many scholars use the abovementioned various methods to describe the residual compressive stress induced by LP to investigate the forming and fatigue resistance performance of aviation components. However, accurately measuring the dynamic deformation behavior for the plates or the integral stiffened plates under ultrahigh strain rates remains challenging during the LPF process of aviation components. Therefore, there is a lack of systematic research on the dynamic deformation behavior and stress evolution process. Furthermore, the underlying mechanism governing dynamic deformation remains unclear and requires further exploration.

In this paper, the parameter optimization for LPF on 6005A–T6 aluminum alloy plates was conducted to explore the constrained deformation of SPs through a combination of simulation and experiment. The impact of multidimensional process parameters on the forming capacity was systematically investigated, while offering a detailed description of the dynamic response and transient behavior of aluminum alloy plates under ultrahigh strain rates. Furthermore, a thorough analysis and comparison were conducted to comprehend the stress evolution process during LPF. Ultimately, by contrasting various LPF schemes for “Plate + Stiffener”, the constrained deformation of SP was comprehended under optimized process conditions. The current work endeavors to explore the dynamic constrained deformation behavior of SP and offers valuable insights for the advancement of LP flexible forming technology.

## 2. Materials and Numerical Analysis

### 2.1. Materials

The lightweight and high-strength 6005A–T6 aluminum alloy was extensively utilized in the skin plates, wing plates, and other SPs found in high-speed transportation equipment, such as aircraft, trains, and subways [25]. The chemical composition of the 6005A-T6 aluminum alloy primarily comprises Fe 0.35, Si 0.9, Mn 0.5, Cr 0.3, Cu 0.3, Ti 0.1, Zn 0.2, Mg 0.7, and Al balanced (wt.%) [26]. Table 1 and Table 2 display the mechanical material data and the processing parameters for LPF, respectively. The LPF treatment parameters in Table 2 are the main technical specifications of the employed laser, including a laser wavelength of 1064 nm, a laser pulse width of 15 ns, and a repetition rate of 2 Hz. Additionally, to standardize the number of LP, the number of tracks is set to 5 times. In this research, the aforementioned experimental data served as the foundation for the finite element simulation, which aimed to analyze the dynamic response of the 6005A-T6 aluminum alloy plate to LP.

### 2.2. Mechanism of LPF

**Figure 1:**
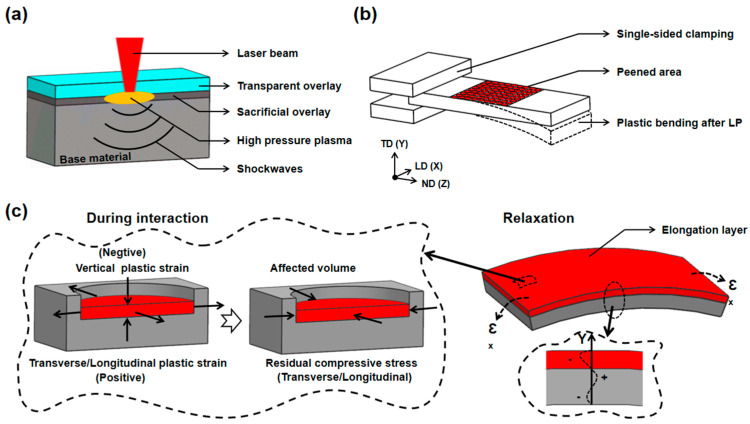
The schematic representation of the LPF process illustrates (**a**) the laser shockwaves, (**b**) the overall bending outcome, and (**c**) the bending mechanism.

Figure 1 depicts the LPF process, which is accomplished by inducing the plastic deformation of the plate through the forceful effect of laser-induced shockwaves [20]. The high-energy short-pulse laser beam irradiation causes the plate surface to instantly vaporize into a high-temperature and high-pressure plasma, and the robust shockwave generated by the plasma explosion propagates towards the interior of the plates [28]. In the depth direction, the stress gradient and the cumulative shock bending moment, induced by the intense shockwave, compel the material into bending deformation, as illustrated in Figure 1a,b. In the process shown in Figure 1c, the intense compression engenders a negative plastic strain in the Y direction. Subsequently, the material undergoes expansion in the X–Z plane, resulting in a positive plastic strain. Upon static relaxation, the confinement imposed by the surrounding material leads to the formation of a residual compressive stress layer on the material surface.

### 2.3. Experimental Design for Parametric Optimization

In an effort to minimize the number of experiments, this study employed the Taguchi method based on the L_9_ orthogonal arrays. These arrays, characterized by unique properties, were chosen from the total number of experiments conducted using the complete factorial method. Three factors with three-level parameters were selected for this study. These factors include laser beam energy (5 J, 8 J, and 11 J), overlap rate (0%, 25%, and 50%), and laser beam diameter (3 mm, 4 mm, and 5 mm), as illustrated in Table 3. Utilizing the designed L_9_ orthogonal array, the experimental parameters designated as S1–S9 in Table 4 were determined.

### 2.4. Pressure Loading Conditions

It is widely recognized that the driving force of LPF originates from the pressure generated by laser-induced shockwaves. As identified by R. Fabbro, the total duration of these laser-induced shockwaves extends 3–5 times the pulse width [29]. Consequently, the shockwave pressure duration of 75 ns is employed for a pulse width of 15 ns. The correlation between the peak pressure of a high-energy laser shockwave, denoted as *P*_max_, and the incident laser power density, represented by *I*_0_, is articulated in Equation (1) [30].

(1) $$P_{\max}=0.01\sqrt{\frac{\alpha }{2\alpha +3}}\cdot \sqrt{Z}\cdot \sqrt{I_{0}}$$

where *α* represents the coefficient for the conversion of internal energy into thermal energy, assigned a value of 0.09, and *Z* denotes the reduced shock impedance [28].

The reduced shock impedance *Z* and the incident laser power density *I*_0_ are indicated in Equations (2) and (3):

(2) $$\frac{2}{Z}=\frac{1}{Z_{1}}+\frac{1}{Z_{2}}$$

(3) $$I_{0}=\frac{\chi \cdot E_{\mathrm{laser}}}{\tau \cdot \pi \cdot {\left(\frac{d}{2}\right)}^{2}}=\frac{4\chi \cdot E_{laser}}{\tau \cdot \pi \cdot d^{2}}$$

where *Z*_1_ represents the confining medium impedance of the water confinement layer (0.165 × 10^6^ g·cm^−2^·s^−1^), and *Z*_2_ displays the target impedance of the 6005A-T6 plate (1.43 × 10^6^ g·cm^−2^·s^−1^). Consequently, the calculated value for *Z* is 0.296 × 10^6^ g·cm^−2^·s^−1^. χ represents the absorption coefficient of the absorber layer, endowed as 0.7, and τ is the laser pulse width, assigned as 15 ns. *E_laser_* denotes the single-pulse laser energy (5 J, 8 J, and 11 J), and *d* displays the laser beam diameter (3 mm, 4 mm, and 5 mm). The computed values for the incident laser power density *I*_0_ and the peak pressure of the shockwaves *P_max_* are attached in Table 4.

**Figure 2:**
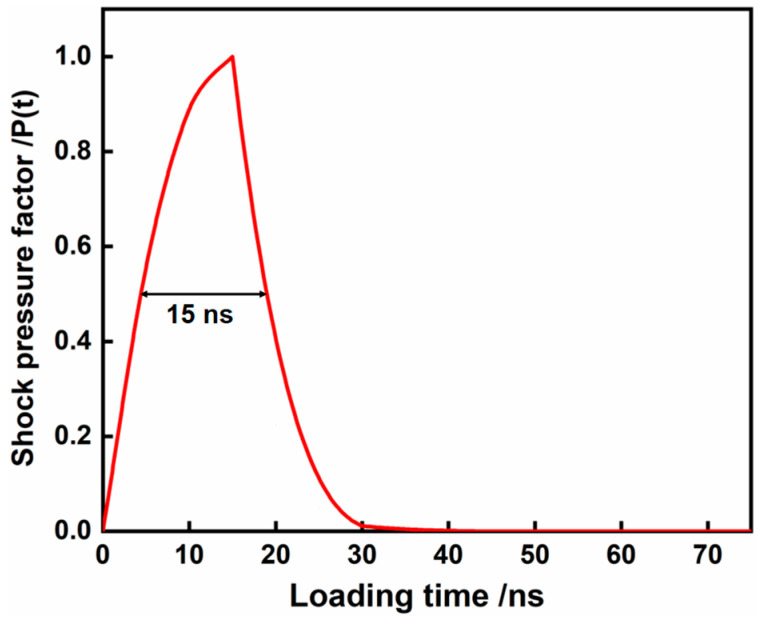
The temporal distribution of shockwave pressure induced by a laser pulse.

The temporal distribution of pressure also considerably influences the outcome of LPF. The evolution of the laser-induced shockwave is bifurcated into the illuminated excitation process and the free decay process. The pressure factor *P*(*t*), as it varies with time *t*, is depicted in Equation (4), and the temporal distribution curve of pressure loading–unloading is illustrated in Figure 2 [31].

(4) $$P(t)=\left\{\begin{array}{ll} -\frac{1}{64}t(t-30) & 0\leq t\leq 15\\ \exp[-0.3(t-15)] & 15\leq t\leq 75 \end{array}\right.$$

### 2.5. Material Constitutive Model

To examine the dynamic response process of 6005A-T6 aluminum alloy under high strain rate plastic deformation, the Johnson–Cook (J–C) constitutive model was employed to simulate the stress evolution induced by laser shockwaves [32]. The J–C model has been widely used in simulating material behavior during the LPF process due to its suitability for complex working conditions with high strain rates and high temperatures. This model, as an empirical model, describes the stress–strain relationship of materials under dynamic loading conditions. It effectively reveals the behavior of materials at high strain rates, especially in LP processes with high strain rates and high stress gradients. Therefore, in the LPF simulation of a plate, the J–C model is preferred due to its wide applicability and relatively mature application experience. The flowing water on the outermost layer of aluminum alloy serves as a constraint layer for pressure rise and cooling. The sub-surface black tape absorbs pulsed laser energy and can be vaporized to form plasma, generating shockwave pressure. The pulsed laser energy does not directly act on the material surface, and the confinement layer and absorption layer protect the aluminum alloy surface from thermal effects. Therefore, this study ignored the influence of laser thermal effects on sample deformation. As a typical cold-worked LPF process, the temperature aspect was excluded from the subsequent simulation. Consequently, the yield stress at a nonzero strain rate $\bar{\sigma }$ can be represented in Equation (5):

(5) $$\bar{\sigma }=(A+B\varepsilon^{n})(1+C\ln{\dot{\varepsilon }}^{*})$$

where ε represents the plastic strain, and ${\dot{\varepsilon }}^{*}$ denotes the normalized plastic strain rate. The parameters *A*, *B*, *C*, *m,* and *n* refer to the initial yield stress, strain hardening modulus, strain rate sensitivity coefficient, thermal softening index, and stress hardening index, respectively. The specific parameter values are presented in Table 5.

### 2.6. Finite Element Method (FEM)

**Figure 3:**
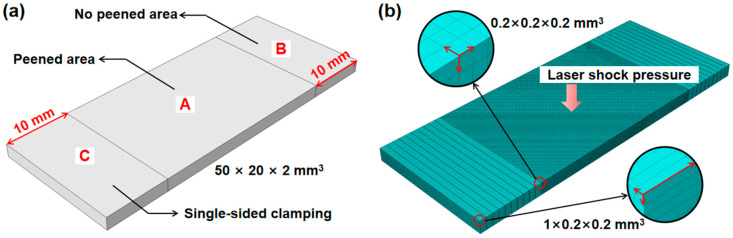
Mesh density division of the peened area and the no peened area. (**a**) three-dimensional dimensions and regional division. (**b**) mesh density division.

The FEM was employed using Abaqus software (Version number: 6.14.4) to simulate each experimental parameter. To reduce computational costs, while achieving more precise outcomes, the areas labeled *A*, *B*, and *C* areas in Figure 3 require division into varying grid densities. A dense mesh density was necessary in the peened area *A* to accurately capture the stress waves. In contrast, the no peened area *B*, which included the clamping area *C*, was assigned a standard mesh density. The length of the peened area *A* (30 mm) was determined by the beam diameter and the overlap rate of the laser spot, ensuring inclusion of the laser spot boundary. For the forming of plates, appropriate clamping methods were adopted to ensure uniform deformation and reduce local stress concentration, thereby improving the forming quality. Usually, the clamping point should be set in a location that does not affect deformation to avoid interference with the forming process. For the forming of plates, the degree of deformation was mainly measured by the length direction, so a fixed length single-sided plate clamping method was adopted. During the simulation process, the fixed constraints were set at one end of the plate model. After the last laser peening, the constraints on the model were removed, and the static rebound was performed. Finally, the residual stress redistribution and the geometrical deformation of the material were obtained. C3D8T–type elements, which are eight-node thermally coupled elements with three-axis displacement capabilities, were employed in this three-area mesh modeling. The total number of elements and nodes for the plate model was 170,000 and 189,981, respectively.

### 2.7. Deformation Characterization of Plates and the Formation Process of SP

**Figure 4:**
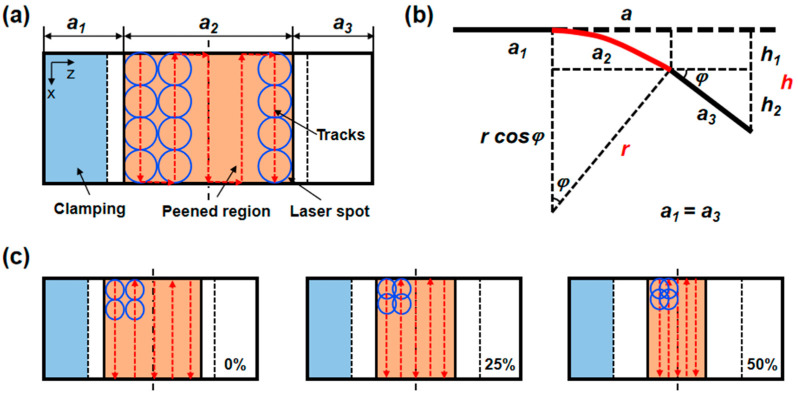
(**a**) Schematic of experimental specimen, (**b**) bending curvature calculation, and (**c**) overlapping trajectories for laser spots of varying sizes.

The variations in overlap rate and beam diameters in the peened area caused the total peened area to change, thereby influencing the length and height of the free end *B*. Hence, the total height displacement value or the total bending curvature could not be solely utilized as indicators of the LP forming capacity. More precisely, following LPF, the bending curvature of the LP region for S1–S9 was employed to characterize the forming capacity. Additionally, considering that the LP area of the plate could be likened to a segment of a sphere, the curvature across this area remained consistent. Consequently, the simplified geometric relationship illustrated in Figure 4b was introduced. Additionally, the overlapping trajectories for laser spots of varying sizes were designed in Figure 4c to ensure a symmetrical distribution in the unfolding direction of forming, as much as possible.

**Figure 5:**
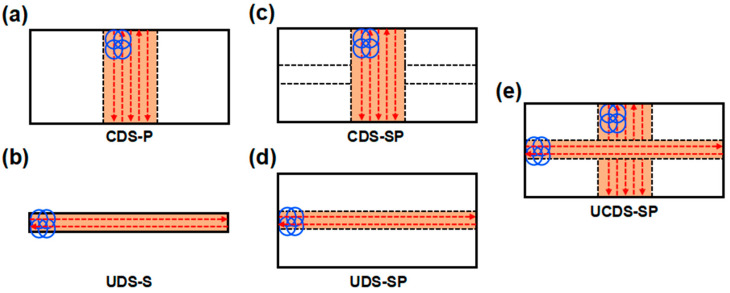
The LPF process of SP. (**a**) CDS–P, (**b**) UDS–S, (**c**) CDS–SP, (**d**) UDS–SP, (**e**) UCDS–SP.

Subsequently, the optimized laser processing parameters (S6: A2B3C1) were employed to investigate the deformation behavior of SP. To facilitate a more effective comparison and comprehension of the deformation situation between the conventional plate and SP, the single plate (S6, chord direction scanning, named CDS–P), and single stiffener (unfolding direction scanning, named UDS–S) were considered separately. The SP was also, respectively, designed as the unfolding direction scanning and chord direction scanning for plates and stiffeners, named CDS–SP and UDS–SP. Furthermore, combining CDS–SP and UDS–SP modes is crucial for complementing each other and enhancing the overall forming capacity and efficiency of SP in two directions. Therefore, a specific design of LPF is depicted in Figure 5, which is primarily categorized into the CDS–P, UDS–S, CDS–SP, UDS–SP, and UCDS–SP. For the forming of single stiffener and stiffened plates, considering the deformation in two directions, a fixed-length single-sided stiffener clamping method with less constraint on the plate was determined.

## 3. Experimental Equipment

**Figure 6:**
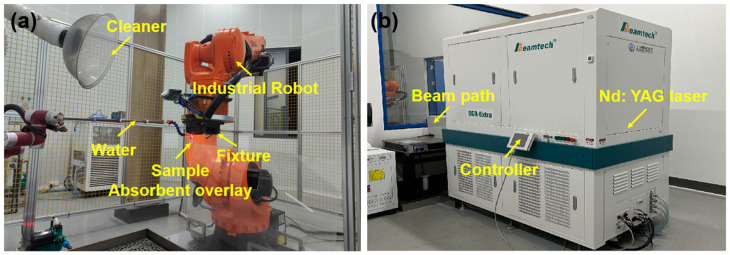
The experimental verification equipment for LP. (**a**) The LP control and motion system; (**b**) the laser.

The experimental verification equipment is displayed in Figure 6. A Q-switched Nd: YAG pulse laser was employed for LP, featuring primary technical specifications, such as a wavelength of 1064 nm, a repetition rate of 5 Hz, and a pulse duration of 15 ns. The experiment was performed in accordance with the laser parameters and the process strategy outlined in Section 2. A 100 μm black tape served as the absorption protective layer, while 1 mm flowing water was exploited as a transparent constraint layer to amplify the shockwave pressure. The plate and SP were secured by fixtures and manipulated using a KUKA robotic arm to facilitate relative spot movement.

The accuracy of the numerical model was verified through experimental steps, such as material preparation, LP experiments, and deformation measurement. This includes using the same or similar materials and conducting LPF experiments based on simulation conditions. The arc height gauge was used to measure the bending deformation and stress–strain distribution of materials. Finally, the experimental data and simulation results were compared to verify the reliability of the model, and the errors between the two were calculated and analyzed.

## 4. Simulating Results and Discussion

### 4.1. Plastic Deformation Progress

**Figure 7:**
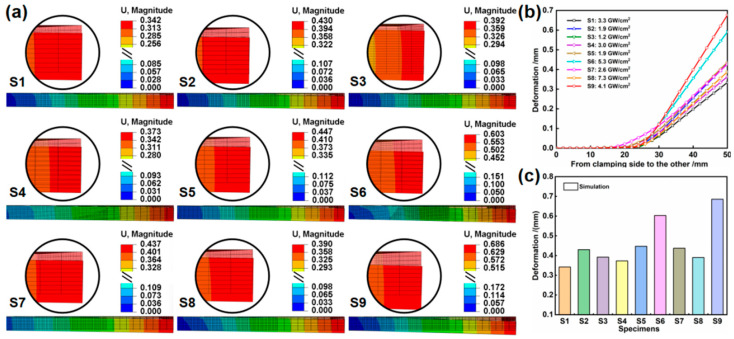
The bending deformation results of medium thickness plates: (**a**) represents the simulation results of the deformation field after static reply, (**b**) depicts the deformation curve, and (**c**) presents the statistical result of the deformation field.

Following Taguchi’s numerical simulation, the deformation field results of the height displacement values and their statistical outcomes for the medium thickness plates after static reply are clearly presented in Figure 7. The residual compressive stresses emerge in the peened area of the medium thickness plate subjected to laser-induced shockwave pressure. To maintain geometric compatibility, the residual compressive stress is partially relieved to generate a negative bending moment that induces tensile deformation in the peened area. The unclamped area undergoes reverse deformation, resulting in a convex curvature. However, varying combinations of the three factors, including beam energy, overlap rate, and beam diameter, each with their corresponding three levels, result in differing total displacement values in the height direction of both the peened area *A* and the free end *B*. Overall, the total displacement values for S6 and S9 are notably large, measuring 0.603 mm and 0.686 mm (S9 > S6), respectively. This outcome is determined by the maximum overlap rate and higher laser power density, while the latter is associated with increasing beam energy and reducing beam diameter.

The bending curvature *ρ* of the peening region *a*_2_ can be determined from the total height displacement value *h* of the plate, as formulated in Equation (6).

(6) $$\left\{\begin{array}{l} h=h_{1}+h_{2}=r-r\cos\varphi +a_{3}\sin\varphi \\ a_{2}=r\varphi \\ \rho =1/r \end{array}\right.$$

where *a*_1_ and *a*_3_, respectively, represent the lengths of the no peened area at the clamping end and the free end, while *a*_2_ denotes the bending length of the peened area under LP action. The parameters *h*_1_, *h*_2_, and *h* present the height displacement values of *a*_2_, *a*_3_, and the *a*_2_ + *a*_3_, respectively. Furthermore, *r* and φ, respectively, represent the bending curvature radius and the corresponding circular center angle of *a*_2_.

**Figure 8:**
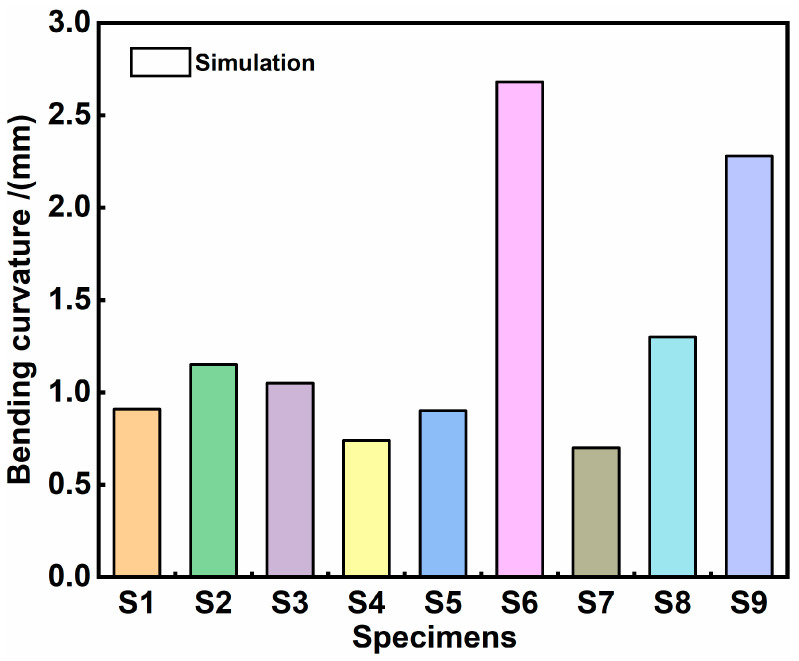
The histogram based on the bending curvature calculations for the peened area, as detailed in Table 6.

The solution outcomes derived from MATLAB (Version number: v6.6.0) code and the corresponding histograms are presented in Table 6 and Figure 8, respectively. Overall, the bending curvature of the peened area aligns closely with the trend in the total deformation height value. However, there are still some variations in the specific bending deformation amplitude. The bending curvature of S6 and S9 is significantly larger than that of the other samples, reaching 2.68 × 10^−3^ mm^−1^ and 2.28 × 10^−3^ mm^−1^, respectively. Compared to S9, S6 displays the maximum bending curvature, which most accurately characterizes the forming ability under three distinct process parameters. This difference is due to the previous bending deformation overlooking the small peened area a_2_, resulting from the overlap of the small laser spot in S6.

**Figure 9:**
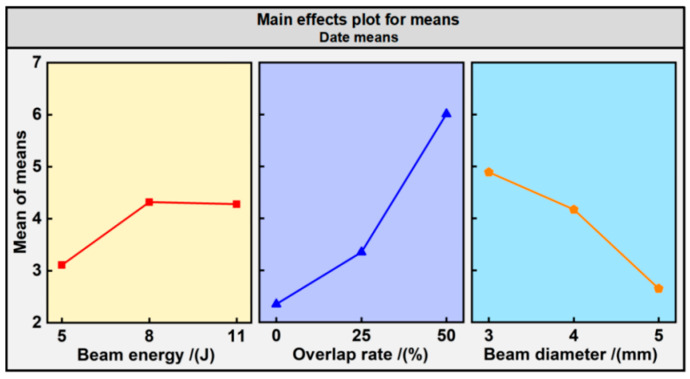
The S/N ratio results.

To further quantify the impact of various process parameters, such as beam energy, overlap rate, and beam diameter on the bending curvature, an analysis of extreme differences calculation was employed. The results of this analysis are presented in Table 7. The signal-to-noise (S/N) ratio was calculated, and the findings can be examined in Figure 9. A comparison of the extreme difference values reveals the following order of influence: overlap rate (R_2_) > beam diameter (R_3_) > beam energy (R_1_). Evidently, the impact degree of the process parameters on the bending curvature is ranked as follows: the largest influence is exerted by the overlap rate, followed by the beam diameter, with the beam energy having the least impact. Consequently, we can ascertain the process parameter design strategy for LPF, simplifying the process design based on the influence degree on the forming curvature. In other words, the overlap rate and beam diameter, employed as the primary variables, facilitate a broad range of deformation adjustments, while the beam energy, served as a secondary variable, achieves more precise deformation adjustments within a narrower range.

From the bending curvature results in the peened area, the bending curvatures of S6 and S9 remain comparatively the largest (S6 > S9). However, the magnitude of the bending curvature values does not correlate with the total displacement values (S9 > S6). The discrepancy arises because smaller-sized samples are selected for the simulation experiments to expedite calculations. As a result, the ratio of the peened area to the unpeened area at the free end (*a*_2_/*a*_3_) is uniformly increased, thereby magnifying the effect of the peened area. In practical applications involving large plates, the ratio of *a_2_* in a small, localized area to *a*_3_ is significantly diminished (*a*_2_/*a*_3_), thereby reducing the effect of the peened area. The larger *a*_2_/*a*_3_ ratios for S6 and S9 (0.44 and 0.63, respectively) lead to an increased magnification degree of the influence and the total displacement value. Therefore, the most accurate representation of the plate’s bending deformation law is not the sum (*h*) of the displacement values of *a*_2_ and *a*_3_ (*h*_1_ and *h*_2_, respectively). Instead, it is the bending curvature *ρ* of the peened region (*a*_2_). This further underscores the correctness and precision of characterizing the LP forming capacity by the bend curvature of the peened area.

### 4.2. Equivalent Plastic Strain

**Figure 10:**
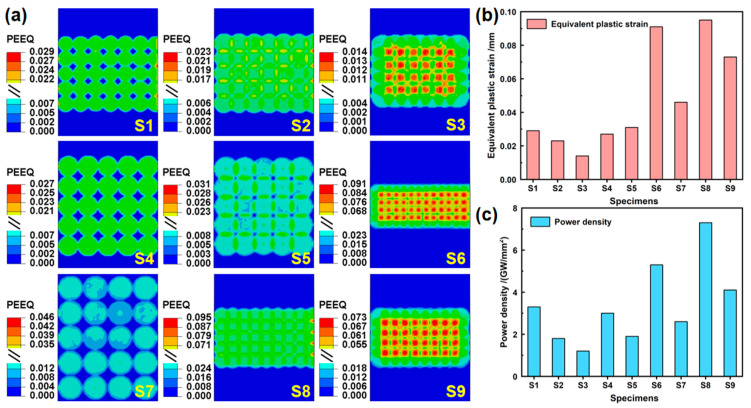
(**a**) Equivalent plastic strains for S1–S9, (**b**) statistical results corresponding to (**a**), and (**c**) the power density.

In Section 4.1, the correctness and precision of characterizing the forming capacity by the bending curvature of the peened area have been effectively demonstrated. However, LPF also encounters challenges related to the forming quality, typically characterized by the equivalent plastic strain as an indicator of influence. The equivalent plastic strains for S1–S9, along with their statistical results, are presented in Figure 10. It is observed that the pattern of variation in the equivalent plastic strain values is positively correlated with laser power density (beam energy/beam diameter). In other words, as the beam energy increases and the beam diameter decreases, the laser power density elevates, leading to larger equivalent plastic strain values and a consequential decline in the forming quality. Furthermore, Section 4.1 highlights that both beam energy and beam diameter are crucial variables that significantly impact the bending curvature degree (forming capacity). Specifically, the increased laser beam energy and reduced laser beam diameter contribute to a larger bending curvature and enhanced forming capacity. Consequently, there exists a competitive mechanism between the forming quality and forming capacity. For this reason, for LPF, we ensure that the relatively larger forming capacity fulfills the application requirements, while concurrently optimizing the forming quality to meet the satisfactory precision standards. For S6 and S9, which exhibit comparatively larger forming capacities (2.68 × 10^−3^ mm^−1^ and 2.28 × 10^−3^ mm^−1^, respectively), there is no substantial disparity in the forming quality (0.091 mm and 0.073 mm, respectively). In summary, the optimal parameter combination, S6 (A2B3C1), is selected.

### 4.3. Stress Evolution

**Figure 11:**
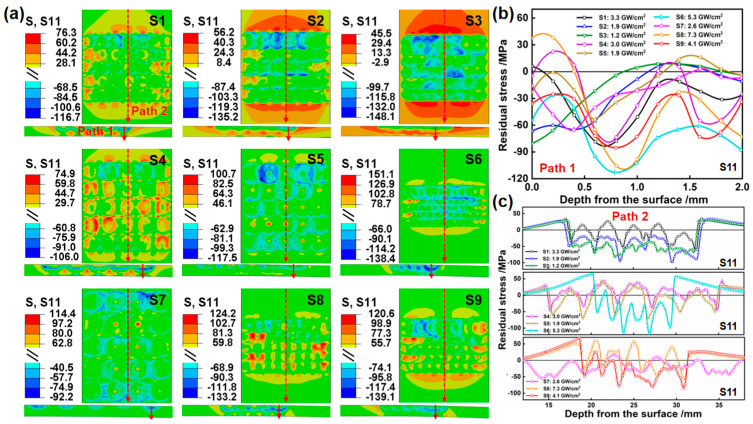
The simulation results of stress field and corresponding stress distribution curve after static reply. (**a**) S11, (**b**) Path 1, and (**c**) Path 2.

**Figure 12:**
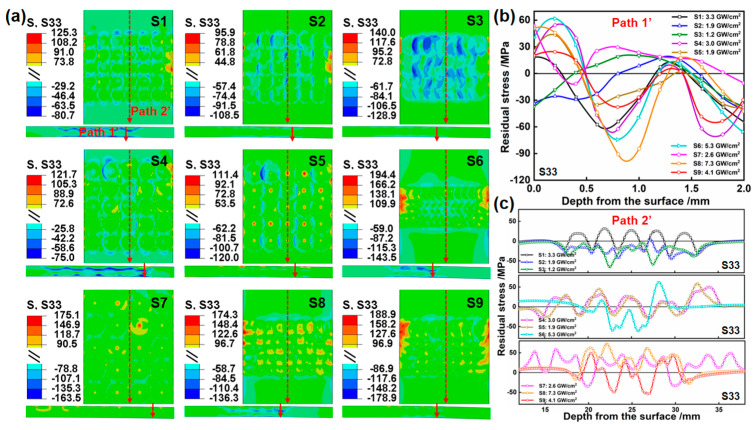
The simulation results of stress field and corresponding stress distribution curve after static reply. (**a**) S33, (**b**) Path 1′, and (**c**) Path 2′.

To gain a deeper insight into the bending deformation and stress distribution of LPF, the stress field after static reply and the stress distribution curve are simulated in Figure 11 and Figure 12. In the cross-sectional area, for multipulse LPF, the stress waves induced by the subsequent pulses overlap with those induced by the preceding pulses. To minimize errors and unify the path, the last LP sequence is selected as Paths 1–1′. In the surface area, it is widely recognized that when stress waves encounter material boundaries, reflection and transmission phenomena occur, the degree of which depends on the physical properties of the materials on both sides of the boundary. To stay as far away as possible from the reflection and transmission areas of the boundary, the center position in the width direction is selected as Paths 2–2′. The stress distribution along Path 1 and Path 1′ typically displays the characteristic pattern of “two-sided compression/middle tension”. Typically, the compressive stress on the upper surface is induced by the compressive deformation resulting from the force effect of LP, while that of the lower surface arises from the equivalent bending moment experienced by the plate following bending deformation. Due to the internal stress balance within the material, tensile stress is observed in the middle region. Moreover, it has been discovered that a larger laser power density induces larger compressive stress and deeper compressive stress layers on the upper surface. A larger laser power density leads to an increased degree of bending deformation, consequently resulting in larger compressive stress and the deeper compressive stress layers in the lower surface. In general, the deeper the compressive stress layers on both surfaces in the depth direction, the shallower the tensile stress in the middle region to balance the compressive stress. Notably, in Figure 11b, samples S1, S6, S8, and S9 of S11 display a penetrating compressive stress, and the reverse side of all samples demonstrates varying degrees of compressive stress. This is attributed to the combined action of large laser power density and significant bending compression. In Figure 11c, insufficient overlap results in the surfaces of samples S1, S4, S5, S7, and S8 experiencing tensile stress. Overall, S6 exhibits the maximum compressive stress. In Figure 12b, compared to S11, the stress of S33, which is perpendicular to the scanning path, is smaller, and there is no occurrence of penetrating compression stress. Similarly, the significant bending deformation in the length direction leads to larger compressive stress on the reverse side of all samples. In Figure 12c, the tensile stress at the surface overlap of samples S1, S4, S5, S7, S8, and S9 are larger, while samples S2, S3, and S6 generally display compressive stress. Among these, S6 possesses the highest stress value.

### 4.4. The Evolution of the Stress and Deformation of S6

**Figure 13:**
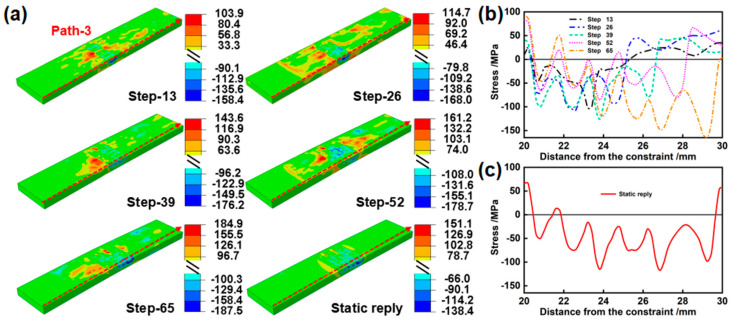
(**a**) Stress evolution in the middle region of S6, (**b**) corresponding stress distribution in the unfolding direction, and (**c**) residual stress distribution after static reply.

To explore the stress transformation during the lap process, the stress evolution simulation for S6 and the corresponding stress distribution curve are presented in Figure 13. When LP is conducted in a single track, a characteristic “middle compression/two-sided tension” stress distribution is observed. With the increase in the number of tracks, compressive stress is induced by the new trajectory couples and expands on the basis of the original compressive stress. Consequently, the compressive stresses in the lap region couple with each other, increasing to 187.5 MPa, while the tensile stress in the edge region also rises to 184.9 MPa. After static reply, compressive stress decreases to −138.4 MPa, exhibiting an overall uniform compressive state.

**Figure 14:**
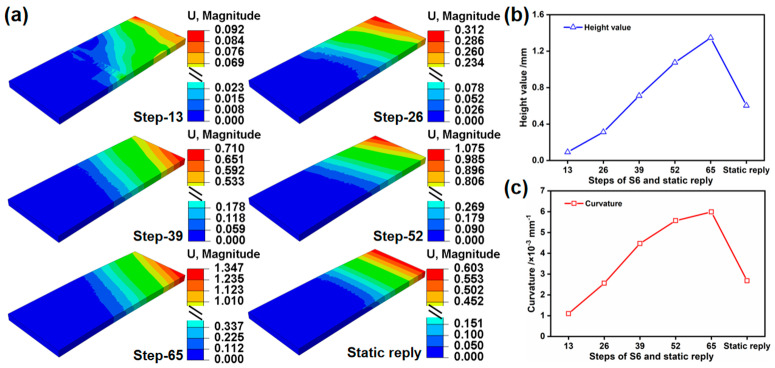
(**a**) Corresponding deformation process illustrated in Figure 13, (**b**) statistical results of the deformation height value in the unfolding direction, and (**c**) corresponding bending curvature.

The deformation evolution process for each step of S6 is depicted in Figure 14, while the deformation value and bending curvature of the peened area are presented in Table 8. As the number of tracks increases, the deformation value exhibits an ascending trend. After five tracks of 65 steps, the maximum deformation attains 1.347 mm. Additionally, the maximum deformation value consistently occurs on the side following the scanning direction. This is due to the force effect of LP causing differences in the oscillation of deformation values at both ends of the plate in the chord direction. Compared to the final 65 steps (1.347 mm), the deformation value after static reply (0.603 mm) reverts by 55.2%, demonstrating forming symmetry in the chord direction. Figure 14b,c displays that, as the number of tracks increases, the incremental amplitude of deformation value and the bending curvature decrease. Following LP, the surface undergoes work hardening, which leads to a reduced plastic flow of the material in the prior direction during subsequent LP processes. Finally, compared to the preceding track, there is a decrease in surface compressive stress and bending deformation.

### 4.5. Deformation Situation and Stress Field of SP

**Figure 15:**
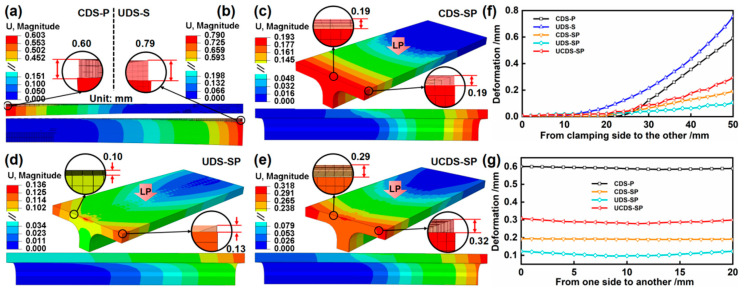
(**a**–**e**) Deformation field after static reply and (**f**,**g**) deformation curves corresponding to (**a**–**e**) in both the unfolding and chord directions.

The simulation results of the deformation field after static reply are presented in Figure 15, and the bending curvature results, calculated according to Equation (6), are displayed in Table 9. It is observed that the height direction yields the deformation value of 0.60 mm and 0.79 mm for the CDS–P (S6) and UDS–S, respectively. However, compared to the calculated bending curvature of the CDS–P (2.68 × 10^−3^ mm^−1^), the larger thickness significantly reduces the bending curvature of the UDS–S (0.63 × 10^−3^ mm^−1^) by an order of magnitude, making deformation considerably more challenging. The bending curvature of the CDS–SP is 0.86 × 10^−3^ mm^−1^. Compared to the CDS–P (2.68 × 10^−3^ mm^−1^), the restriction imposed by the stiffener increases the forming difficulty by approximately 3.1 times under identical LP conditions. Similarly, the bending curvature of the UDS–SP is 0.08 × 10^−3^ mm^−1^. Compared to the UDS–S (0.63 × 10^−3^ mm^−1^), the restraint imposed by the plate amplifies the forming difficulty by approximately 7.9 times. The results reveal that the plate and stiffener of SP impose constraints on each other, presenting significant challenges to bend forming. Under nearly the same number of laser spots (65 and 66, respectively), the bending curvature of the CDS–SP is larger than that of the UDS–SP (0.86 × 10^−3^ mm^−1^ and 0.08 × 10^−3^ mm^−1^, respectively, approximately 10.8 times), reflecting a larger forming capacity. In contrast, the former induces local bending deformation in the middle of the peened area, while the latter generates the overall deformation from the clamping side to the opposite end. There are no significant disparities in the deformation value of the CDS–SP in the middle and on both sides, which measures 0.19 mm. Similarly, the same conclusion can be deduced from Figure 15g. It is noted that there are discrepancies in the chord direction deformation values of the UDS–SP, exhibiting the “middle smaller (0.10 mm)/two-sided larger (0.13 mm)” phenomenon. The deformation in the middle area of the plate is significantly less than the two-sided area. The constraint of the stiffener increases the thickness of the middle area, and the bending deformation is more difficult. Moreover, the constraint effect of the stiffener on the bending deformation is larger than the positive bending moment formed by the inertia effect, so the deformation on two sides of the plate is more significant. Consequently, the UDS–SP is more predisposed to achieving efficient two-way forming. The results illustrate that the aforementioned two forming processes hold significant importance for LPF, primarily encompassing the forming capacity and efficiency. The CDS–SP possesses a larger forming ability and is more concentrated on large-sized forming in a single unfolding direction; whereas, the UDS–SP offers more advantages in achieving efficient integrated forming in two directions. The bidirectional forming of the plate and stiffener is termed the UCDS–SP. By amalgamating the advantages of the two aforementioned modes, the deformation height values in the unfolding and chord directions increase to 0.291 mm and 0.318 mm, respectively. These values are marginally smaller than the sum of the aforesaid two modes, which are 0.295 mm (0.193 + 0.102 mm) and 0.329 mm (0.193 + 0.136 mm). This is attributed to the overlapping area between the two peened regions, and the plastic deformation that occurs at this area after the first LPF constrains the forming value of the subsequent LP.

**Figure 16:**
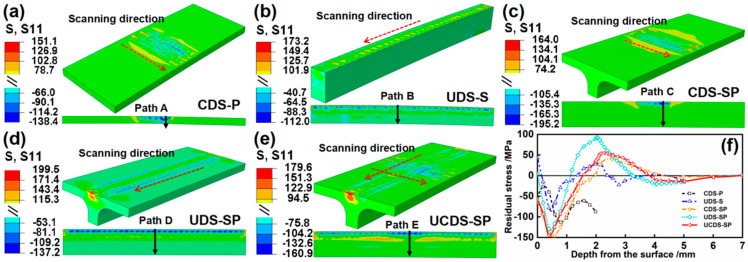
(**a**–**e**) Stress fields of S11 after static reply and (**f**) corresponding stress distribution curve along Paths A–E.

**Figure 17:**
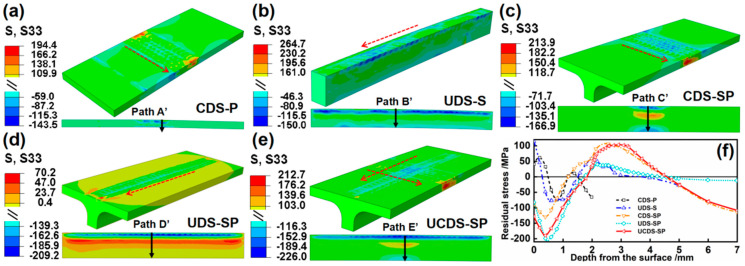
(**a**–**e**) Stress fields of S33 after static reply and (**f**) corresponding stress distribution curve along Paths A’–E’.

The simulation results of the stress field after static reply are presented in Figure 16 and Figure 17. Compared to the CDS–P, the large thickness gives rise to a distinct stress field. The stress distribution of the UDS–S in the depth direction consistently exhibits a “two-sided compression/middle tension” pattern, with no occurrence of penetrating compressive stress observed. The subsurface layer consists of a high-density residual compressive stress layer, and the compressive stress of S33 (−150.0 MPa) is larger than that of S11 (−112.0 MPa). This discrepancy is ascribed to the more significant plastic deformation of S33 compared to S11, which leads to a more significant rebound effect and consequently larger residual stress. Furthermore, Figure 17f illustrates that the large bending deformation induces more substantial compressive stress on the material’s backside in the length direction (S33) compared to the width direction (S11). The stress field of CDS–SP differs from that of plate. The stiffener extends the propagation distance of the stress wave in the depth direction. Consequently, the stress balance position shifts downward, while the stress field distribution of the plate remains unaltered. Additionally, it is noted that the compressive stress of S11 (−195.2 MPa) is larger than that of S33 (−166.9 MPa). In contrast to the unfolding (Z) direction forming constrained by the stiffener, the plate’s plastic forming in the chord (X) direction, which faces lesser constraints, is more feasible, causing more significant rebound effects and larger residual stress. However, regarding the stiffener, the large bending deformation in the Z direction leads to severe compression on the stiffener’s backside, presenting a larger compressive stress of S33 (−103.4 MPa). The stress field of the UDS–SP closely resembles that of the UDS–S. The subsurface layer in the depth direction is also characterized by a high-density compressive stress layer, uniformly exhibiting a “two-sided compression/middle tension” stress distribution. Variously, the presence of the plate enlarges the propagation range of stress waves to all directions, which aids in the bidirectional deformation of the plate and elevates the compressive stress after static rebound (from −112.0 MPa to −137.2 MPa of S11 and from −150.0 MPa to −209.2 MPa of S33). The compressive stress of S33 (−209.2 MPa) is larger than that of S11 (−137.2 MPa). The compressive stresses of the UCDS–SP of S11 and S33, respectively, increase to −160.9 MPa and −226.0 MPa, primarily in the overlapping area of the two peened regions.

## 5. Experimental Verification of Simulation Results

**Figure 18:**
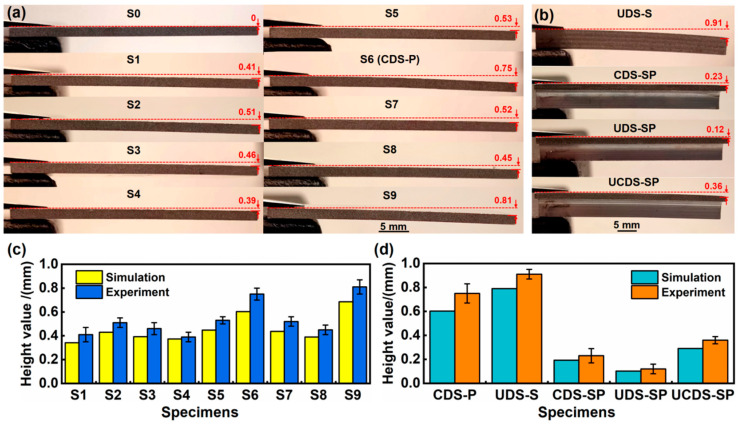
The bending deformation results and statistical results of height displacement values for conventional plates, stiffener, and SP after LP experiments. (**a**) Plates, (**b**) UDS–S, CDS–SP, UDS–SP, and UCDS–SP, (**c**,**d**) the statistical results of (**a**,**b**).

To facilitate a direct comparison with simulation outcomes, the LP experiment follows the procedure detailed in the preceding simulation, as illustrated in Figure 18. It is widely recognized that bending deformation is dependent on both positive bending moments, induced by force effects, and negative bending moments, resulting from stress gradients [31]. In Figure 18a, it is observed that all samples manifest varying degrees of overall convex bending deformation, which highlights the significant influence of negative bending moments induced by stress gradients. Furthermore, as depicted in Figure 18c,d, the experimental results of S1–S9 plates, stiffener, and SP closely match the simulation outcomes. Significantly, the experimental values consistently surpass the simulation values, albeit by a slight margin. This pattern suggests a slight variance between experimental observations and simulated predictions. It is necessary to consider that the minor thermal effects disregarded during the simulation process could inherently induce a certain degree of softening in the 6005A–T6 aluminum alloy material. Conversely, it is also essential to recognize that the complex interaction among the constraint layer, absorption layer, and the material itself, as observed in actual experimental conditions, tends to be significantly more intricate than what is commonly simulated. This discrepancy in complexity inevitably leads to the discrepancies between the values obtained through the experiment methods and those derived from the simulation approach.

The relative percentage error is a common measure of the degree of deviation between the simulation and experimental results. The uncertainty is described through relative error analysis to evaluate the accuracy of the model predictions. By comparing the actual measurement results with the model prediction results, the relative errors were obtained, as displayed in Table 10. The relative error range of the two is roughly between 4.4% and 19.6%. For engineering applications, the relative error is acceptable in a range of less than 20%. Therefore, reasonable error ranges prove and verify the effectiveness of the model. The uncertainty comes from multiple aspects, such as model establishment (model selection, material parameter estimation), numerical errors (mesh partitioning and time step), boundary condition selection, and measurement errors.

## 6. Conclusions

This paper conducted the parameter optimization for LPF on 6005A–T6 aluminum alloy plates to explore the constrained deformation of SP. Specifically, the following conclusions are drawn:

(1) The impact of process parameters on bending curvature is ranked as follows: the overlap rate is the largest, followed by the beam diameter; the beam energy is the smallest. Therefore, adjusting the overlap rate and beam diameter of the primary variables obtains a wide range of deformations, while the beam energy of secondary variable achieves a more precise deformation.

(2) During the 12–track LPF process, there is an overall upward trend in deformation values and a dynamic increase in bend curvature. After static relaxation, there is a recovery of 55.2% in the deformation value compared to the final bending curvature.

(3) The plate and stiffener of the SP are mutually constrained, severely limiting the overall formation. The CDS–SP exhibits a greater forming capacity than that of the UDS–SP, and there are no significant disparities in the CDS–SP deformation in both the middle and side regions. However, discrepancies exist in the chord direction deformation of the UDS-SP, which shows a pattern of “smaller at middle/larger at two sides”.

(4) The CDS–SP exhibits a larger bending curvature than the UDS–SP, indicating a superior forming capacity. The CDS–SP focuses more on large-sized forming along a single unfolding direction, while the UDS–SP excels at achieving efficient, integrated two-way forming.

## Figures and Tables

**Table 1:** The mechanical material data used for simulations [27].

Property	Value
Density/Kg/m^3^	2700
Young modulus/GPa	69
Poisson’s ratio	0.31
Tensile strength/MPa	290
Yield strength/MPa	264
Elongation/%	10

**Table 2:** The LPF treatment parameters [27].

Processing Parameters	Value
Laser wavelength/nm	1064
Laser pulse width/ns	15
Repetition rate/Hz	2
Number of tracks/times	5

**Table 3:** The Taguchi parameters and levels utilized to optimize the laser forming process.

No.	Parameters	Symbol	Level 1 (A)	Level 2 (B)	Level 3 (C)
1	Beam energy	J	5	0%	3
2	Overlap rate	times	8	25%	4
3	Beam diameter	mm	11	50%	5

**Table 4:** The parameters of the designed experiments.

Design Point Number	Beam Energy/J	Overlap Rate/%	Beam Diameter/mm	Power Density/GW·cm^−2^	Peak Pressure/MPa
S1	5	0%	3	3.3	1662
S2	5	25%	4	1.8	1228
S3	5	50%	5	1.2	1003
S4	8	0%	4	3.0	1585
S5	8	25%	5	1.9	1261
S6	8	50%	3	5.3	2107
S7	11	0%	5	2.6	1476
S8	11	25%	3	7.3	2466
S9	11	50%	4	4.1	1853

**Table 5:** J–C constitutive model parameters.

Processing Parameters	Value
*A*	264
*B*	313
*C*	0.0029
*n*	0.553
*m*	1.7
Fusion temperature	605
Initial temperature	20

**Table 6:** The solution results calculated by MATLAB code for the peened area.

No.	*a*_1_ = *a*_3_/mm	*a*_2_/mm	*h*/mm	*ψ*/rad	*h*_2_/mm	*h*_1_/mm	*r*/mm	*ρ*/×10^−3^ mm^−1^
S1	17.5	15	0.342	0.0137	0.240	0.102	1094.9	0.91
S2	17.5	15	0.430	0.0172	0.301	0.129	872.1	1.15
S3	17.5	15	0.392	0.0157	0.275	0.117	955.4	1.05
S4	15	20	0.373	0.0149	0.224	0.149	1342.3	0.74
S5	15	20	0.447	0.0179	0.268	0.179	1117.3	0.90
S6	20.5	9	0.603	0.0241	0.494	0.108	373.4	2.68
S7	12.5	25	0.437	0.0175	0.218	0.219	1428.6	0.70
S8	19	12	0.390	0.0156	0.296	0.094	769.2	1.30
S9	19	12	0.686	0.0274	0.522	0.164	438.0	2.28

**Table 7:** The results derived from the extreme difference calculation include the S/N ratio values for various parameters at different levels, along with their effectiveness ranking.

No.	Beam Energy/J	Overlap Rate/Times	Beam Diameter/mm	Bend Curvature *ρ*/×10^−3^ mm^−1^
S1	5	0%	3	0.91
S2	5	25%	4	1.15
S3	5	50%	5	1.05
S4	8	0%	4	0.74
S5	8	25%	5	0.90
S6	8	50%	3	2.68
S7	11	0%	5	0.70
S8	11	25%	3	1.30
S9	11	50%	4	2.28
Sum of index 1	3.11	2.35	4.89	Total/(T) = 11.71
Sum of index 2	4.32	3.35	4.17	
Sum of index 3	4.28	6.01	2.65	
Range/(R)	R_1_ = 1.21	R_2_ = 3.66	R_3_ = 2.24	
Effectiveness ranking	3	1	2	R_2_ > R_3_ > R_1_

**Table 8:** The solution results computed using MATLAB code.

No.	*a*_1_/mm	*a*_2_/mm	*a*_3_/mm	*h*/mm	*φ*/rad	*r*/mm	*ρ*/×10^−3^ mm^−1^
Step–13	20.5	3	26.5	0.092	0.0033	909.1	1.10
Step–26	20.5	4.5	25	0.312	0.0115	391.3	2.56
Step–39	20.5	6	23.5	0.710	0.0268	223.9	4.47
Step–52	20.5	7.5	22	1.075	0.0418	179.4	5.57
Step–65	20.5	9	20.5	1.347	0.0539	167.0	5.99
Static reply	20.5	9	20.5	0.603	0.0241	373.4	2.68

**Table 9:** The statistical data of deformation value and bending curvature.

Forming Methods	*a*_1_ = *a*_3_/mm	*a*_2_/mm	*H*/mm	*ψ*/rad	*r*/mm	*ρ*/×10^−3^ mm^−1^
CDS-P (S6)	20.5	9	0.603	0.0241	373.4	2.68
UDS-S	0	50	0.790	0.0316	1582.3	0.63
CDS-SP	20.5	9	0.193	0.0077	1168.8	0.86
UDS-SP	0	50	0.102	0.0041	12,195.1	0.08
UCDS-SP	/	/	0.291	/	/	/

**Table 10:** The relative error obtained by comparing actual measurement results with model prediction results.

No.	Simulation/mm	Experiment/mm	Error/%	No.	Simulation/mm	Experiment/mm	Error/%
S1	0.342	0.41	16.7	S8	0.390	0.45	13.3
S2	0.430	0.51	15.7	S9	0.686	0.81	15.3
S3	0.392	0.46	14.8	CDS–P	0.603	0.75	19.6
S4	0.373	0.39	4.4	UDS–S	0.790	0.91	13.2
S5	0.447	0.53	15.7	CDS–SP	0.193	0.23	16.1
S6	0.603	0.75	19.6	UDS–SP	0.102	0.12	15.0
S7	0.437	0.52	16.0	UCDS–SP	0.291	0.36	19.2

## Data Availability

The original contributions presented in the study are included in the article, further inquiries can be directed to the corresponding author.
